# Collaborative harm reduction efforts lead to the first detection of 5-cyano isotodesnitazene in illicit street drugs

**DOI:** 10.1038/s41598-026-35256-4

**Published:** 2026-01-13

**Authors:** Bárbara F. C. Barra, Joana R. P. Pereira, Daniela R. Ferreira, Hans Oeri, Daniel Martins, Rui M. Almeida, Giles Oatley, Nuno R. Neng, Helena Gaspar, Alexandre Quintas

**Affiliations:** 1 Laboratório de Ciências Forenses e Psicológicas Egas Moniz, Quinta da Granja, Caparica, Almada, 2829 − 511 Portugal; 2 Centro de Química Estrutural, Institute of Molecular Sciences, Departamento de Química e Bioquímica, Faculdade de Ciências, Universidade de Lisboa, Lisboa, 1749-016 Portugal; 3 Kosmicare, Rua Cesário Verde 17, Lisboa, 1170-090 Portugal; 4 BioISI-Biosystems & Integrative Sciences Institute, Departamento de Química e Bioquímica, Faculdade de Ciências, Universidade de Lisboa, Campo Grande, Lisboa, 1749-016 Portugal; 5 Kykeon Analytics Ltd., Plaza Nuestra Señora del Buen Camino number 11, planta BJ, Puerta C, Madrid, 28023 Spain; 6 Egas Moniz Center for Interdisciplinary Research (CiiEM); Egas Moniz School of Health & Science, Quinta da Granja, Caparica, Almada, 2829 − 511 Portugal; 7 Institute of Innovation, Science and Sustainability, Department of Information Systems, Federation University, Victoria, 3805 Australia

**Keywords:** Nitazenes, GC-MS/MS, NMR, HRMS, Drug-checking services, Synthetic opioids, Chemistry, Drug discovery

## Abstract

In the ongoing opioid crisis, regulatory restrictions on fentanyl and its derivatives have led to the emergence of nitazenes, a class of highly potent synthetic opioids synthesised during the 1950s but never approved for medical use. Structurally distinct from traditional opioids, nitazenes have gained increasing presence in illicit drug markets since 2019, contributing to a growing number of drug-related deaths across Europe, North America, and Australia. The rapid proliferation of novel nitazene analogues presents a significant challenge to law enforcement and public health systems. Drug-checking services (DCS) are currently one of the most effective tool for early detection and response, enabling real-time surveillance and consequently reducing the impact of overdoses due to Novel Synthetic Opioids. As a complement, universities can provide the scientific expertise and infrastructure necessary to confirm molecules detected by DCS. This study is the first report identifying and confirming 5-cyano isotodesnitazene in a street-sold drug, through an international collaboration involving two DCSs and three academic institutions. The structural elucidation was achieved through complementary analytical techniques comprising gas chromatography, mass spectrometry, and nuclear magnetic resonance spectroscopy. The present work highlights the essential role that cross-sector cooperation and analytical innovation play in tackling the rapidly evolving threat of novel synthetic opioids.

**Supplementary Information:**

The online version contains supplementary material available at 10.1038/s41598-026-35256-4.

## Introduction

**Fig. 1:**
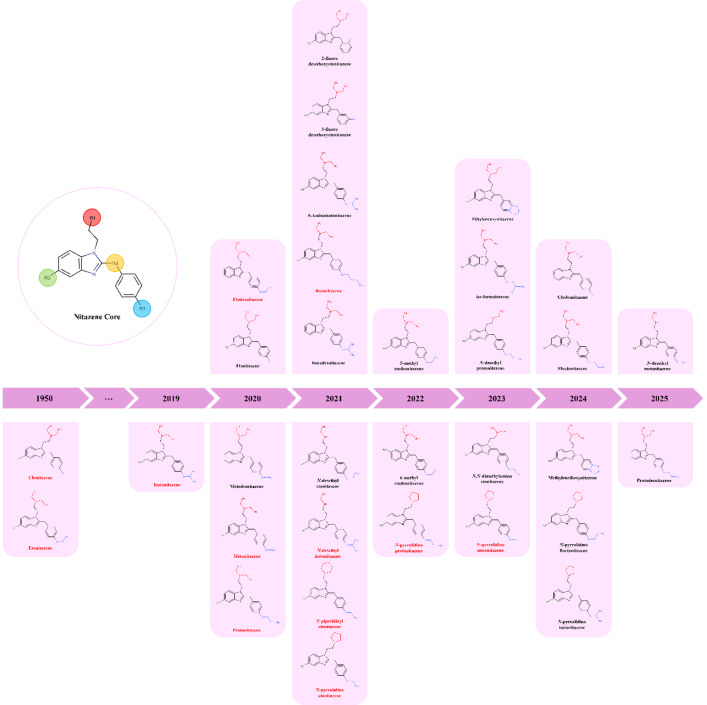
Chemical structures of etonitazene and clonitazene, the earliest nitazenes, together with 30 other nitazene analogues reported to UNODC-EWA from 2019 to 2025^6,7^. Compounds shown in red are currently subject to international drug control conventions under the United Nations.

In the current opioid crisis, the restrictions applied to fentanyl and its derivatives have led to the emergence of nitazenes, a class of synthetic opioids first synthesised in the mid-1950s^1,2^ as a pain-relieving alternative to morphine. In the late 1950 s, etonitazene was a target of preclinical research in rats and rhesus monkeys, but it never progressed to clinical trials due to an unfavourable balance between therapeutic and toxic effects^3,4^. Chemically, nitazenes differ from the typical morphine-like phenanthrene motif and from meperidine analogues such as fentanyl. This class of compounds is derived from a 2-benzylbenzimidazole core structure through the addition of various substituents. The structures of etonitazene and clonitazene, the first two nitazenes placed under international control in 1961, along with the 30 nitazenes recently reported through the Early Warning Advisory (EWA), shown in Fig. 1^5^, indicate that these compounds typically feature an alkylamino substituent at R1, a nitro group in position R2 (C5 from benzimidazole core), and an alkoxy group in the *para-*position of the benzyl ring (R3). Based on the type of amino substituent at R1, nitazenes can be grouped into five structural subclasses: (i) *N*,*N*-dimethyl derivatives; (ii) *N*,*N*-diethyl derivatives; (iii) *N*-desethyl analogues, bearing an *N*-ethyl group; (iv) nitazepynes with a pyrrolidinyl group and; (v) nitazepipnes with a piperidinyl group. Nitazenes lacking a substituent in position R2 are designated as desnitro‑nitazenes or simply desnitazenes. Moreover, the substituent in position R2 can also be a methyl (CH_3_) or an amino (NH_2_) group. Some monofluorinated nitazenes have also been identified, with the fluorine atom located at the *ortho*, *meta*, or *para* position of the benzyl ring, or incorporated into the alkoxy substituent in R3. A chlorine atom can also be present as a substituent on the benzyl ring. Modifications at the benzylic carbon (R4) have already been described in the literature^1^, and it is expected that new nitazenes may emerge on the illicit drug market with structural variations at this position. Due to the novelty of this NPS class, common nitazene names have not yet been harmonised within the scientific community. Thus, Table S1 in Supplementary Material compiles the common names along with their IUPAC names, the year each compound was first reported to the United Nations Office on Drugs and Crime Early Warning Advisory (UNODC-EWA) and, where applicable, the year it was scheduled under the 1961 Single Convention on Narcotic Drugs. This overview allows for temporal analysis of nitazene emergence, highlighting the monitoring role of the UNODC-EWA in identifying New Psychoactive Substances (NPS), and illustrating the subsequent international legal control measures.

The emergence of nitazenes in the illicit drug market, during the current opioid wave, dates from 2019, when isotonitazene was detected in Canada and Estonia (March and April 2019, respectively)^8,9^. One year later, metonitazene and protonitazene were identified in postmortem toxicological analyses of drug-related deaths^10^. Since then, the death toll resulting from the use of nitazene-containing opioids has been on the rise, according to data provided by countries monitoring these Novel Synthetic Opioids (NSO) and data from scientific publications analysing samples from DCSs, seizures, and wastewaters^11–16^. Briefly, nitazenes were found in 29% and 48% of opioid-related deaths during 2023 in Estonia and Latvia, respectively^10^. Also in 2023, inadvertent misuse of nitazenes as heroin resulted in multiple overdoses and localised poisoning outbreaks in Ireland^17^ and France^10^. In 2024, nitazenes were involved in 179 deaths in the United Kingdom, in some cases, more than one nitazene was found in postmortem toxicological analysis^18^. In the United States, from 2020 to 2023, the number of deaths by opioids surpassed 300,000, and anecdotal reports estimate that nitazenes have been involved in 2,000 deaths^19^. During the same period, sentinel toxico-surveillance in Australian and Victorian emergency departments confirmed the presence of protonitazene, metonitazene, isotonitazene, butonitazene, etodesnitazene and *N*-pyrrolidino etonitazene across 32 cases^20^. As a consequence of the numerous overdose cases associated with emerging nitazenes, isotonitazene, metonitazene, protonitazene, *N*-pyrrolidino etonitazene, etodesnitazene, butonitazene, *N*-desethyl isotonitazene, *N*-pyrrolidino metonitazene, *N*-pyrrolidino protonitazene, and *N*-piperidinyl etonitazene, Fig. 1, were scheduled under the 1961 Convention on Narcotic Drugs between 2021 and 2025 (see Table S1, Supplementary Material)^7,21^.

Recently, the European Union Drugs Agency (EUDA) highlighted the urgent need for enhanced data collection on nitazenes, citing “the potential of these substances to impact negatively on Europe’s public health in the future”^22^. In the current year, the UNODC-EWA on NPS reported that nitazenes have spread globally. Given the dynamic nature of the NSO market, the dissemination of new compounds within this group of synthetic opioids is highly likely^23–25^. Moreover, drug control laws in many countries are typically substance-specific, and the rapid and continuous appearance of novel compounds on the street market poses significant challenges for regulatory and law enforcement agencies^26,27^. The swift creation of structural analogues of controlled substances can quickly outpace existing legal frameworks, rendering them inadequate for timely control and enforcement^28^. DCS have emerged as a critical harm‑reduction intervention, providing timely chemical analysis of unregulated drug supplies during the last global synthetic-opioid overdose crisis. DCSs inform drug users about the contents of their substances, but also serve as sentinel systems supporting broader detection, monitoring, and public health response efforts^29^. For instance, in early 2024, nitazene test strips enabled the rapid detection of potent analogues, such as isotonitazene, thereby enhancing the ability of DCSs to identify high-risk synthetic opioids at the point of care^30^. In Australia, a DCS detected the unexpected presence of *N*-pyrrolidino isotonitazene in a counterfeit oxycodone tablet, triggering collaborative action with academic laboratories^31^. Mobile and fixed-site DCS initiatives have demonstrated how integration with syringe service programs and academic partnerships can deliver real-time surveillance data that inform public health interventions and regulatory responses^32^. These examples illustrate the individual and system-level value of DCS in providing early warning alerts regarding novel nitazenes. Nevertheless, the high potency of nitazenes and their potential public harm calls for a more robust national and international drug threat response framework that expands drug-checking. Without coordinated action, the rapid emergence of novel opioids, including nitazenes, will continue to outpace existing public health and legal systems. Promoting cross‑sector collaborations and implementing standardised drug-checking systems at scale will assist policy-makers to the rapid identification of NSOs.

The present work stems from an international collaboration between two DCSs and three academic institutions aimed at advancing harm reduction efforts to address the NSO issue. In this collaborative enterprise, upon detecting a potential NSO candidate, the DCS forwarded the sample for analysis using gas chromatography-triple quadrupole mass spectrometry (GC-MS/MS). Analysis of the mass spectra fragmentation patterns indicated the presence of a potential unknown nitazene, while quadrupole time-of-flight high-resolution mass spectrometry (HRMS) enabled the determination of its molecular formula. Structural elucidation was subsequently attained using nuclear magnetic resonance (NMR) spectroscopy, confirming unequivocally the presence of 5-cyano isotodesnitazene. The entire process was completed in under two weeks, supporting the added value of the DCS-Academia partnership in identifying NPS hitting the streets without reference standards.

## Materials and methods

### Drug sample

The suspect sample (a white powder) was submitted for analysis by Kykeon’s drug-checking laboratory to Kosmicare and the LCFPEM within the scope of the LCFPEM, Kosmicare and Kykeon collaboration protocol.

### Reagents and chemicals

For GC-MS/MS analysis, HPLC-grade methanol (MeOH, > 99.9%) was purchased from Honeywell (Riedel-de-Haën, Germany) and *N*‑trimethylsilyl‑*N*‑methyltrifluoroacetamide (MSTFA, 95–100%) from Macherey-Nagel (Germany). For HRMS analysis, methanol and acetonitrile (LC-MS grade, HiPerSolv CHROMANORM^®^, VWR Chemicals/Avantor), formic acid (Optima™ LC/MS grade, Fisher Chemical, Thermo Fisher Scientific), and ultrapure water purified using a Milli-Q Direct system (resistivity ≥ 18.2 MΩ·cm, Millipore) were used. For NMR analysis, dimethyl sulfoxide-D6 (DMSO-*d*_6_, D, 99.9%) from Cambridge Isotope Laboratories (United States of America) was used.

### Sample preparation

For GC-MS/MS analysis, 1 mg of the sample was dissolved in 200 µL of MeOH (final concentration of 5 mg/mL) and vortexed for 1 min. For HRMS analysis, this solution was diluted in methanol to a final concentration of 0.05 mg/mL. To assess the presence of additional compounds, the remaining GC-MS/MS solution of 5 mg/mL was dried under a stream of nitrogen (99.9992%, Praxair, USA), followed by the addition of 50 µL of MSTFA. The mixture was then heated in a microwave oven (700 W, Selecline, France) for 3 min, followed by GC-MS/MS analysis. Additionally, 2 mg of the sample was dissolved in 200 µL of DMSO-*d*_6_ (final concentration of 10 mg/mL) to acquire the NMR spectra.

### Instrumentation

The analysis by GC-MS/MS was performed on an Agilent 7000E Triple Quadrupole GC-MS Detector with GC 8890 and 7693 A Automatic Liquid Sampler (ALS) system (Santa Clara, CA, USA). The GC separation was performed on an Agilent HP-5MS UI column (30 m × 0.25 mm × 0.25 μm). The oven temperature programme started at 240 °C and was held for 1 min, then increased by 30 °C/min until it reached 300 °C, which was maintained for 15 min. Helium was used as the carrier gas at a flow rate of 1.5 mL/min. The sample was injected in split mode (50:1) at 280 °C and the injection volume was 1 µL. The transfer line temperature was set to 300 °C, the ion source temperature was set to 250 °C, and the quadrupole temperature was set to 150 °C. The mass spectrometer electron ionisation was set to 70 eV, and the run was performed in scan mode over the *m/z* range of 55–550. The compound’s mass spectrum was identified using the NIST MS Search (v3.0). For precursor and product ion, the capillary voltage was 20 eV.

The HRMS analysis was performed by direct injection of 1 µL of the nitazene sample solution (0.050 mg/mL), via the LC system (Elute™ UPLC, Bruker Daltonics) with constant flow of 300 µL/min (solvent mixture of acetonitrile/water (1:1, v/v), both containing 0.1% formic acid), coupled to the mass spectrometer (Impact II UHR–QqTOF, Bruker Daltonics) with electrospray ionisation (ESI). Data acquisition was performed in positive ionisation mode at a spectra rate of 1 Hz, over a mass range of 40–1200 m/z. Operating parameters were: capillary voltage 4500 V, nebuliser pressure 0.4 bar, drying gas flow rate 4.0 L/min, and drying temperature of 180 °C. MS data were acquired using an auto MS/MS method with a 3 s cycle time. Active exclusion was triggered after three spectra and released after 0.3 min. Precursor reconsideration was applied if the ratio of current to previous intensity was up to 5, with an absolute fragmentation threshold of 48 counts. Collision energy was set to 15 eV.

Spectra were externally calibrated via direct infusion of 1 µM sodium formate-acetate adducts using high pressure syringe pump (JAYTEE, NE-1010) at 180 µL/h. MS files were calibrated using Compass Data Analysis 6.3 (Bruker Daltonics) in high-precision calibration mode, achieving a standard deviation of < 0.137 ppm for calibration points.

Data processing and interpretation of full-scan MS and MS/MS spectra were carried out using Compass Data Analysis 6.3 (Bruker Daltonics). Accurate mass measurements were obtained for both precursor and fragment ions, and molecular formula assignments were performed with the SmartFormula tool (Compass DataAnalysis 6.3, Bruker Daltonics), applying accurate mass and isotopic pattern matching.

NMR spectra were acquired at 298 K using a Bruker Ascend 500 MHz spectrometer operating at 500.13 MHz for ^1^H NMR and 125.61 MHz for ^13^C APT NMR, equipped with a Prodigy triple resonance cryogenic probe CRPN2—TR-^1^H&^19^F/^13^C/^15^N-5 mm-EZ (Bruker Instruments, Rheinstetten, Germany). The sample solution was placed in a 3 mm NMR tube. Chemical shifts (δ) were expressed as parts per million (ppm) and referenced to the signal of DMSO-*d*_6_ (δ^1^H = 2.50 ppm, δ^13^C = 39.52 ppm). Coupling constants *(J*) were reported in Hertz (Hz) units. Structure elucidation was achieved through the respective assignment of carbon and proton signals based on the analysis of NMR spectra obtained using 1D (^1^H and ^13^C APT) and 2D ([^1^H-^1^H]-COSY, [^1^-^13^ C]-HSQC-ed, and [^13^C-^1^H]-HMBC) techniques. To assist the resonance assignment, a 2D [^1^H-^1^H]-NOESY experiment was acquired, using a mixing time of 400 ms, with a resolution of 4096 (*t*_*2*_) × 512 (*t*_*1*_) data points, covering a sweep width of 5.5 kHz in the ^1^H dimension, with 2 scans per increment.

## Results and discussions

### GC-MS/MS analysis

The suspected nitazene powder sample was analysed by GC-MS/MS, revealing a single peak with a retention time of 8.099 min. Three nitazene analogues were suggested by the NIST MS Library, namely protonitazene, isotonitazene, and butonitazene. The comparison of the obtained mass spectrum (Fig. S1 in Supplementary Material) with the library spectra showed that the three main and most intense peaks at *m/z* 58, 86 (base peak), and 107 matched those of the reference compounds. However, secondary peaks at *m/z* 156, 231, 260, and 262 did not correspond to any of the library entries. To further investigate, multiple reaction monitoring mode was used to identify the parent ions of *m*/*z* 58, 86, 107, and 260. A precursor ion at *m/z* 390 was identified, suggesting the presence of a different nitazene analogue, probably with a molecular mass of 390 g/mol. This molecular mass was later confirmed by HRMS analysis.

After searching the Cayman Chemical library, two compounds with the same molecular weight were found (5-cyano protodesnitazene and 5-cyano isotodesnitazene). As observed previously, subtle differences in the mass spectrum, namely the absence of the *m/z* 156 and 231 fragments, suggest that the unknown sample is most likely 5-cyano isotodesnitazene. Subsequent NMR analysis (*vide infra*) confirmed the presence of this nitazene along with citric acid in approximately equimolar amounts in the sample. For further confirmation, the nitazene sample was derivatised with MSTFA, followed by GC-MS/MS analysis. The results confirmed the presence of citrate and methyl citrate in the derivatised sample. No other substances or interferences were observed in the GC-MS/MS, HRMS, or NMR analyses.

**Fig. 2:**
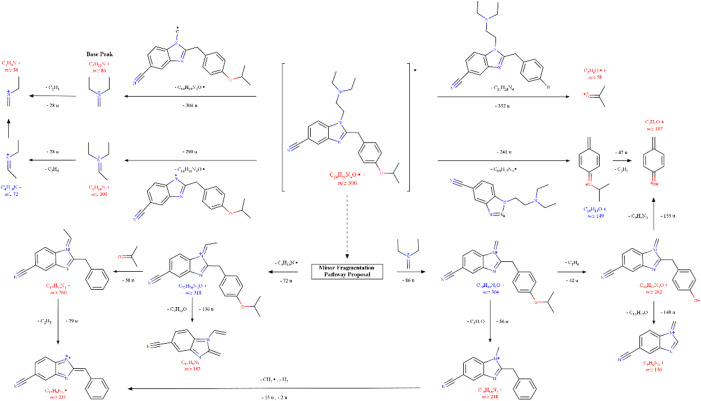
Proposed fragmentation pathways of 5-cyano isotodesnitazene.

In fact, a detailed analysis of the mass spectrum of the new nitazene (Fig. S1) reveals a fragmentation pattern consistent with the main characteristic fragmentation pathways of nitazene analogues described in the literature^33,34^, as shown in Fig. 2. These include α-cleavage at the amine group at R1 (Fig. 1), resulting in the formation of an iminium ion, which is commonly observed as the base peak. For all nitazenes bearing a *N*,*N*-diethylamino group, this ion appears at *m/z* 86, as is the case with our nitazene analogue. This fragment can subsequently undergo the elimination of an ethyl group to yield the *m/z* 58 ion.

Additionally, the *m/z* 58 ion may also be formed from the cleavage of the isopropoxy group attached to the benzyl moiety, resulting in the formation of the acetone radical ion. The *m/z* 107 ion is formed through benzylic cleavage, followed by elimination of the substituent on the oxygen atom as a neutral prop-1-ene molecule. Another common fragment ion is *m/z* 100, corresponding to the cleavage of the methylene linker attached to the benzimidazole core (N1).

Figure 2 also shows the proposed minor fragmentation pathways to the formation of smaller characteristic ions of 5-cyano isotodesnitazene, such as *m/z* 262, 260, 248, 231,182, and 156. The α-cleavage at the amine atom (N1) of the 5-cyano-benzimidazole, also generates a 5-cyano-1-methylenebenzimidazole core linked to the benzyl group (*m/z* 304) ion. Subsequent loss of prop-1-ene leads to the formation of the *m/z* 262 ion. Further cleavage at the benzyl group results into *m/z* 156 ion, corresponding to the 5-cyano-1-methylenebenzimidazole ion. However, an alternative cleavage of the fragment at *m/z* 304 involving the elimination of the isopropoxy group can produce the *m/z* 248 ion. In accordance with the MS/MS results, the *m/z* 231 ion can be formed from the *m/z* 248 ion through the loss of 17 u, which suggests the elimination of the N1 methyl group and a H_2_ molecule as the proposed pathways. On the other pathway, inductive cleavage at the nitrogen atom of the *N*,*N*-diethylamino group may generate a 5-cyano-1-ethylidenebenzimidazole core linked to the benzyl group with *m/z* 318 ion. The subsequent elimination of acetone generates the *m/z* 260 ion, and further loss of the N1 ethyl group results in the *m/z* 231 ion. If cleavage of the *m/z* 318 ion occurs at the benzyl group, it may produce the *m/z* 182 ion via an aromatic rearrangement, as proposed by Vandeputte et al.^35^. Interestingly, this ion was not originated from the *m/z* 260 or *m/z* 231 fragments, as confirmed through precursor/product ion analysis by GC-MS/MS.

### HRMS analysis

The accurate mass spectrum (Fig. S2a) of the nitazene sample obtained through HRMS displays two ion peaks: a major peak at *m*/*z* 391.2485, compatible with the protonated molecule of 5-cyano isotodesnitazene, C_24_H_31_N_4_O (calcd. 391.2492; error + 1.9 ppm), and a minor peak at *m*/*z* 781.4900, assigned to C_48_H_61_N_8_O_2_ (calcd. 781.4912; error + 1.6 ppm), representing [2 M + H]⁺. This allows to establish unambiguously its molecular formula as C_24_H_31_N_4_O. In the tandem mass spectrum of the protonated molecule (Fig. S2b), the presence of the *N*,*N*-diethyl substituent in this nitazene analogue can be confirmed by the base peak fragment ion at *m/z* 100.1113 (calcd. 100.1121; error + 7.3 ppm), corresponding to the 1,1-diethylaziridinium ion (Fig. S2c). This iminium ion, resulting from the α-cleavage of the amino group at R1, is characteristic of nitazene derivatives containing an *N*-diethyl moiety at the R1^35,36^. Additionally, two minor peaks are observed at *m/z* 107.0485 (error + 6.3 ppm) and *m/z* 276.1122 (error + 3.3 ppm). This latter peak results from the simultaneous loss of diethylamine and prop-1-ene from the protonated 5-cyano-isotodesnitazene molecule. The ion at *m/z* 107 is usually observed in nitazene derivatives containing an alkoxy moiety at R3^35,37^.

### NMR structural characterisation

**Fig. 3:**
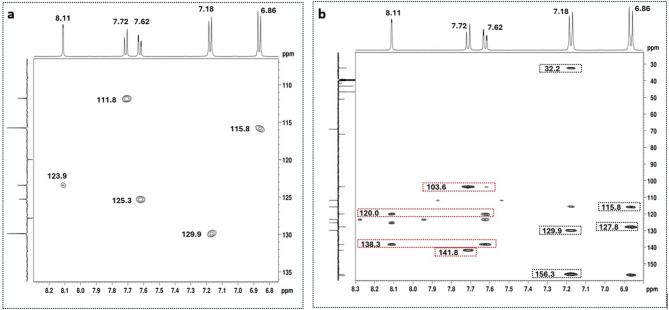
[^1^-^13^ C]-HSQC-ed (**a**), and [^13^C-^1^H]-HMBC (**b**) spectra (DMSO-*d*_*6*_) of the nitazene sample, zoomed in to the regions containing the key correlations of aromatic protons of 2-benzylbenzimidazole moiety.

The ¹H NMR spectrum in the aromatic region (Fig. S3) of the suspected nitazene sample revealed the presence of an AMX spin system, comprising three aromatic proton signals at 7.72 ppm (1 H, *d*, *J* = 8.4 Hz), 7.62 ppm (1 H, *dd*, *J* = 8.4, 1.5 Hz), and 8.11 ppm (1 H, *d*, *J* = 1.1 Hz), as well as an AA′BB′ system consisting of two apparent doublets at 6.86 ppm (2 H, *J* = 8.7 Hz) and 7.18 ppm (2 H, *J* = 8.7 Hz). Additionally, in the aliphatic region (Fig. S4), a singlet at 4.30 ppm attributable to two equivalent benzylic protons was observed, along with an AX_6_ spin system characteristic of an isopropoxy group at 4.55 ppm (1 H, *sept*, *J* = 6.0 Hz) and 1.23 ppm (6 H, *d*, *J* = 6.0 Hz). These spin systems were corroborated by the correlations observed in the [¹H–¹H] COSY spectrum (Fig. S5). The corresponding ^13^C APT signals (Fig. S6 and Fig. S7) were assigned based on one-bond ¹H–¹³C correlations observed in the HSQC-ed spectrum (Fig. 3a). All these spectral features are consistent with the presence of a trisubstituted benzimidazole ring with an isopropoxybenzyl group at C2.

**Fig. 4:**
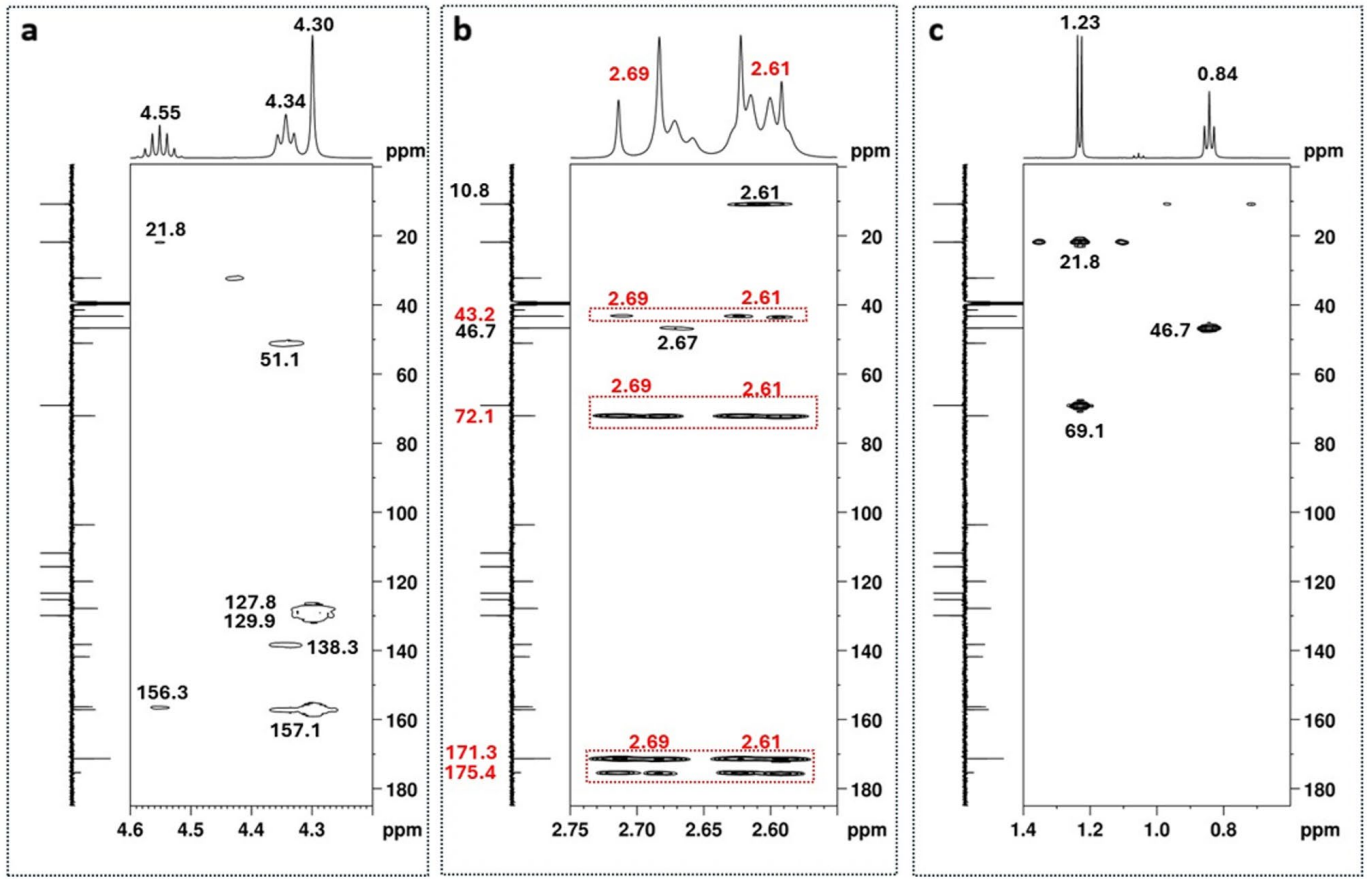
[^13^C-^1^H]-HMBC spectra (DMSO-*d*_*6*_) of the nitazene sample expanded in the regions of the key correlations of aliphatic protons: (**a**) 4.20–4.6 ppm; (**b**) 2.55–2.75 ppm; (**c**) 0.70–1.5 ppm.

The presence of a *para*-isopropoxybenzyl substituent was further confirmed through the following key HMBC correlations: i) the 3-bond correlations of the aromatic protons of the AA’BB’ system with the quaternary aromatic carbons at 127.8 and 156.3 ppm (Fig. 3b), as well as with the methine aromatic carbons at 115.8 and 129.9 ppm (Fig. 3b), support the presence of a 1,2-disubstituted aromatic ring; ii) the 3-bond correlation between the methine proton at 4.55 ppm and the carbon at 156.3 ppm (Fig. 4a) indicates that the isopropoxy group is one of the aromatic substituents, allowing this quaternary carbon to be assigned to position C5’’; iii) the correlation of the two equivalent protons at 4.30 ppm with the aromatic carbons at 127.8 ppm and 129.9 ppm (Fig. 4a) confirms that these protons are, in fact, benzylic, allowing those carbon signals to be assigned to carbons C2’’ and C3’’, and consequently, the signal at 115.8 ppm to C4’’. The four-bond COSY correlation between the benzylic protons (4.30 ppm, H1’’) and the aromatic proton at 7.18 ppm (H3’’) (Fig. S5) also supports the structural assignment of the 1,4-disubstituted aromatic ring. The 2-bond HMBC correlation (Fig. 4a) observed between the benzylic protons at 4.30 ppm (H1’’) and the quaternary carbon at 157.1 ppm (C2) attested that the isopropoxy group is the substituent at position C2 of the benzimidazole ring.

The ^1^H NMR spectrum (Fig. S4) also shows three spin systems that partially overlap in the 2.75–2.55 ppm region, as revealed by the HSQC spectrum (Fig. S7). Within this region, which integrates to a total of 10 protons, two doublets at 2.69 and 2.61 ppm were identified, displaying a geminal coupling constant of 15.4 Hz. These signals are characteristic of an AB system comprising two diastereotopic protons that show a HSQC correlation with the carbon signal at 43.2 ppm (Fig. S7). The HMBC correlations of these protons (Fig. 4b in red) with the carbons at 43.2, 72.1, 171.3, and 175.4 ppm confirmed their assignment to a citric acid moiety (Table 1). These findings are consistent with NMR data of an authentic citric acid sample. Following the identification of this compound, it was inferred that the remaining six proton signals in the 2.75–2.55 ppm region correspond to the nitazene analogue.

The presence of a diethylamino group can be readily identified by: (i) the three-bond COSY correlation between the triplet at 0.84 ppm (6 H, *J* = 7.2 Hz) and the quartet at 2.61 ppm (*J* = 7.1 Hz) (Fig. S5); (ii) this quartet overlaps in the ^1^H NMR spectrum with the proton signal at 2.61 ppm (2 H, d, *J* = 15.4 Hz) of citric acid (Fig. S4 and S7). The total integration of the two signals is 6 H, indicating that the quartet integrates to 4 H, which supports the presence of two equivalent CH_2_ groups; (iii) The corresponding carbon signal at 46.7 ppm (C4’), characteristic of a methylene group bound to a nitrogen atom, has a HMBC correlation with the proton signals at 2.61 and 0.84 ppm (Fig. 4b and c); (iv) The carbon at 10.8 ppm (C5’) of the two equivalent methyl groups at 0.84 ppm (H5’) correlates in the HMBC spectrum with the methylene protons at 2.61 ppm (H4’) (Fig. 4b).

The remaining ^1^H signal corresponds to a A_*2*_ × _2_ spin system consisting of the triplet at 4.34 ppm (2H, *J* = 6.7 Hz) that correlates in COSY with the triplet at 2.67 ppm (*J* = 6.7 Hz) (Fig. S5). This triplet overlaps with the proton signal of the citric acid at 2.69 ppm (2H, d, *J* = 15.4 Hz) (Fig. S4 and Fig. S7). The total integration of the two signals is 4H, showing that the triplet at 2.67 ppm integrates to 2H (Fig. S4). The chemical shifts of the corresponding carbon of the two methylene groups of the A_*2*_ × _2_ spin system appear at 41.4 and 51.1 ppm (Fig. S7). The 3-bond HMBC correlations of the carbon at 46.7 ppm (C4’) with the triplet at 2.67 ppm (Fig. 4b) enabled the carbons at 41.4 and 51.1 to be assigned to positions C1’ and C2’, respectively.

The 3-bond HMBC correlation observed between the methylene protons at 4.34 (H1’) with the quaternary carbons 157.1 (C2) and 138.3 ppm (Fig. 4b), together with their NOESY correlation, with the aromatic proton at 7.72 ppm (Fig. S8), indicate that the *N*,* N*-diethylethan-1-amine group is linked to the benzimidazole ring at the N1. This allows the assignment of the signal at 7.72 ppm to the C7 position, and the quaternary carbon at 138.3 ppm to position C7a. Consequently, the remaining aromatic methine groups with ^13^C/^1^H chemical shifts at 7.62/125.7 ppm and 8.11/123.9 ppm can be assigned to positions C6 and C4, respectively. This structural assignment confirms that the third substituent is linked to C5.Finally, the presence of a nitrile group at C-5 could be attested by three key HMBC 3-bond correlations between AMX protons and the quaternary carbons (Fig. 3b): (i) H4 (8.11 ppm) with 138.3 (C7a) and 120.0 ppm; (ii) H7 (7.72) with 103.6 and 141.8 ppm; (iii) H6 (7.62 ppm) with 120.0 and 138.3 (C7a) ppm. These findings support the assignment of the carbon signal at 103.6 ppm to C5, the signal at 141.8 ppm to C3a, and the signal at 120.0 ppm to the nitrile carbon.

All these NMR findings allowed the unambiguous assignment of all ^1^H and ^13^C NMR signals, summarised in Table 1, and confirmed the presence of both 5-cyano isotodesnitazene and citric acid in the sample. Although some overlap occurs between their proton signals, integration of signals in the ^1^H NMR spectrum (Fig. S3 and Fig. S4) indicates that the two compounds are present in approximately equimolar amounts.

**Table 1:** NMR spectroscopic data (DMSO-*d*_*6*_)^a^ of 5-cyano isotodesnitazene and citric acid.


**Position**	**Type**	**δ** ^**13**^**C**	**δ** ^**1**^**H**, ***m***, ***J*** **(Hz)**	**COSY**	**HMBC**
	**5-cyano isotodesnitazene**				
**2**	Cq (-)	157.1	-	-	4.30, 4.34
**3a**	Cq (-)	141.8	-	-	7.71
**4**	CH (+)	123.4	8.11 (1 H, *d*, 1.1)	7.62	7.62
**5**	Cq (-)	103.6	-	-	7.72
**6**	CH (+)	125.3	7.62 (1 H, *dd*, 1.5, 8.4)	8.11, 7.72	8.11
**7**	CH (+)	111.8	7.72 (1 H, *d*, 8.4)	7.62	-
**7a**	Cq (-)	138.3	-	-	8.11, 7.62, 4.34
**CN**	Cq (-)	120.0	-	-	8.11,7.62
**1’**	CH_2_ (-)	41.4	4.34 (2 H, *t*, 6.7)	2.67	2.67
**2’**	CH_2_ (-)	51.1	2.67 (2 H, *t*, 6.7)*	4.34	2.61,4.34
**4’**	CH_2_ (-)	46.7	2.61 (4 H, *q*, 7.2)*	0.84	2.61, 2.67, 0.84
**5’**	CH_3_ (+)	10.8	0.84 (6 H, t, 7.1)	2.61	2.61
**1"**	CH_2_ (-)	32.2	4.30 (2 H, *s*)	7.18	7.18
**2"**	Cq (-)	127.8	-		6.86
**3"**	2x CH (+)	129.9	7.18 (2 H, *d*, 8.7)	6.86, 4.30	4.30. 7.18
**4"**	2x CH (+)	115.8	6.86 (2 H, *d*, 8.7)	7.18	6.86, 7.18
**5"**	Cq (-)	156.3	-		7.18
**7"**	CH (+)	69.1	4.55 (1 H, *sept*, 6.0)	1.23	1.23
**8"**	2x CH_3_ (+)	21.8	1.23 (6 H, *d*, 6.1)	4.55	1.23
	**Citric moiety**				
**1**	2x Cq (-)	171.3	-		2.61, 2.69
**2**	2x CH_2_ (-)	43.2	2.69 (2 H, *d*, 15.3)		2.61, 2.69
			2.61 (2 H, *d*, 15.3)*		
**3**	Cq (-)	72.1	-		2.61, 2.69
**4**	Cq (-)	175.4	-		2.61, 2.69

^a^ Assignments are supported by 2D-NMR experiments: COSY, HSQC, HMBC, * overlapped total integration 10 H.

### The role of forensic intelligence

Forensic scientists from Australia^38^ recently presented a ‘call to arms’ for forensic laboratories, policing, academia and supporting industries alike to implement and entrench forensic intelligence. They stated that now is the time to build foundations towards this future in academia, and to develop partnerships and facilitate delivery for supporting industries. Forensic intelligence, or the proactive use of forensic data, is being used in this case to detect emerging drug threats and develop Early Warning Systems (EWS).

Various programs exist worldwide, including the following: Center for Forensic Science Research & Education (CFSRE) ‘s NPS Discovery program launched in 2018, funded by the U.S. National Institute of Justice (NIJ)^39^; Emerging Drugs Network of Australia (EDNA/EDNAV), a toxicosurveillance system using clinical and toxicological data to detect emerging drug threats^40,41^; European Union Drug Agency (EUDA) EWS^42,43^, with the EWA Tox-Portal tool to collect, analyse and share data on toxicology and harm related to the use of NPS; The UNODC-EWA provides information on NPS including trend data, chemical details on individual substances and supporting documentation on laboratory analysis^5^.

## Conclusions

The detection and identification of NPSs remains a significant challenge for public health and forensic networks, particularly when seized or submitted samples lack prior identification and are absent from available spectral libraries. In such cases, as demonstrated in this study, comprehensive structural elucidation requires the application of multiple complementary analytical techniques, including GC-MS/MS, HRMS, and NMR. These methods enable accurate mass measurements, fragmentation patterns analysis to infer key molecular characteristics, and NMR-based structural information on chemical environments, supporting confident compound identification in the absence of reference standards.

In this study, the combined application of the aforementioned analytical techniques allows the unequivocal structural elucidation of 5-cyano isotodesnitazene from a drug checking sample, in the absence of reference materials.

This methodological approach was developed in response to a real-world drug-checking case that required the collaboration of multiple laboratories at an international level. This study exemplifies the value of close collaboration between academic institutions and DCS organisations. Such partnerships bridge scientific expertise with frontline public health needs, enabling the timely identification of emerging synthetic drugs and improving risk communication to communities.

The work presented here constitutes not only a technical advancement with its less-than-two weeks turnaround but also demonstrates how coordinated cross-sector collaboration between academia and supporting industries can generate timely forensic intelligence to protect public health, inform policy, and strengthen the societal response to synthetic drug use.

## Supplementary Information

Below is the link to the electronic supplementary material.

Supplementary Material 1

## Data Availability

The correspondent author (AQ) will provide relevant data upon reasonable request.
