# Supplementary material for: Collaborative harm reduction efforts lead to the first detection of 5-cyano isotodesnitazene in illicit street drugs
Source: Sci Rep. 2026 Jan 13;16:5163. doi: 10.1038/s41598-026-35256-4 (PMC12881620; doi:10.1038/s41598-026-35256-4)
Supplement: Supplementary file 1 — Supplementary Material 1 [file 41598_2026_35256_MOESM1_ESM.docx]

**Supplementary material**

**Collaborative Harm Reduction Efforts Lead to the First Detection of 5-Cyano Isotodesnitazene in Illicit Street Drugs**

**Bárbara F. C. Barra ^a,b,c,†^, Joana R. P. Pereira ^a,b,†^, Daniela R. Ferreira ^a,b,d^, Hans Oeri ^e^, Daniel Martins ^c^, Rui M. Almeida ^a,f^, Giles Oatley ^g^, Nuno R. Neng ^a,b,f^, Helena Gaspar ^d^, Alexandre Quintas ^a,f,*^**

^a^ Laboratório de Ciências Forenses e Psicológicas Egas Moniz, Quinta da Granja, 2829-511 Caparica, Almada, Portugal.

^b^ Centro de Química Estrutural, Institute of Molecular Sciences, Departamento de Química e Bioquímica, Faculdade de Ciências, Universidade de Lisboa, 1749-016 Lisboa, Portugal.

^c^ Kosmicare, Rua Cesário Verde 17, 1170-090 Lisboa, Portugal.

^d^ BioISI-Biosystems & Integrative Sciences Institute, Departamento de Química e Bioquímica, Faculdade de Ciências, Universidade de Lisboa, Campo Grande, 1749-016 Lisboa, Portugal.

^e^ Kykeon Analytics Ltd., Plaza Nuestra Señora del Buen Camino number 11, planta BJ, Puerta C, 28023 Madrid, Spain.

^f^ Egas Moniz Center for Interdisciplinary Research, Egas Moniz School of Health & Science, Quinta da Granja, 2829-511 Caparica, Almada, Portugal.

^g^ Institute of Innovation, Science and Sustainability, Department of Information Systems, Federation University, Victoria, 3805, Australia.

* Corresponding author: alexandrequintas@gmail.com (A. Quintas)

**^†^** Authors contributed equally; All authors have read and agreed to the published version of the manuscript.

**Tables**

| Common name | Synonyms | IUPAC name | InChI keys | UNODC EWA | 1961 UN Convention |
| --- | --- | --- | --- | --- | --- |
| Etonitazene | - | 2-(2-(4-ethoxybenzyl)-5-nitro-1*H*-benzo[d]imidazol-1-yl)-*N,N*-diethylethan-1-amine | PXDBZSCGSQSKST-UHFFFAOYSA-N | - | 1961 |
| Clonitazene | - | 2-(2-(4-chlorobenzyl)-5-nitro-1*H*-benzo[d]imidazol-1-yl)-*N,N*-diethylethan-1-amine | GPZLDQAEBHTMPG-UHFFFAOYSA-N | - | 1961 |
| Isotonitazene | - | *N,N*-diethyl-2-(2-(4-isopropoxybenzyl)-5-nitro-1*H*-benzo[d]imidazol-1-yl)ethan-1-amine | OIOQREYBGDAYGT-UHFFFAOYSA-N | 2019 | 2021 |
| Etodesnitazene | Etazene,  Desnitroetonitazene | 2-(2-(4-ethoxybenzyl)-1*H*-benzo[d]imidazol-1-yl)-*N,N*-diethylethan-1-amine | WMZWQRKNBMKEOO-UHFFFAOYSA-N | 2020 | 2023 |
| Flunitazene | Fluonitazene,  4-Fluoro desethoxyetonitazene | *N,N*-diethyl-2-(2-(4-fluorobenzyl)-5-nitro-1*H*-benzo[d]imidazol-1-yl)ethan-1-amine | OKEXRSNRQKCYRB-UHFFFAOYSA-N | 2020 | - |
| Metodesnitazene | Desnitrometonitazene, Metazene | *N,N*-diethyl-2-(2-(4-methoxybenzyl)-1*H*-benzo[d]imidazol-1-yl)ethan-1-amine | IOQZPMOVWNEIKR-UHFFFAOYSA-N | 2020 | - |
| Metonitazene | - | *N,N*-diethyl-2-(2-(4-methoxybenzyl)-5-nitro-1*H*-benzo[d]imidazol-1-yl)ethan-1-amine | HNGZTLMRQTVPBH-UHFFFAOYSA-N | 2020 | 2022 |
| Protonitazene | - | *N,N*-diethyl-2-(5-nitro-2-(4-propoxybenzyl)-1*H*-benzo[d]imidazol-1-yl)ethan-1-amine | SJHUJFHOXYDSJY-UHFFFAOYSA-N | 2020 | 2023 |
| 2-Fluoro desethoxyetonitazene | - | N,N-diethyl-2-(2-(3-fluorobenzyl)-5-nitro-1H-benzo[d]imidazol-1-yl)ethan-1-amine | OKEXRSNRQKCYRB-UHFFFAOYSA-N | 2021 | - |
| 3-Fluoro desethoxyetonitazene | - | N,N-diethyl-2-(2-(3-fluorobenzyl)-5-nitro-1H-benzo[d]imidazol-1-yl)ethan-1-amine | WTQYDOAOYZERJJ-UHFFFAOYSA-N | 2021 | - |
| 5-Aminoisotonitazene | - | 1-(2-(diethylamino)ethyl)-2-(4-isopropoxybenzyl)-1*H*-benzo[d]imidazol-5-amine | DVCBBZBNZSSXMS-UHFFFAOYSA-N | 2021 | - |
| Butonitazene | Butoxynitazene | 2-(2-(4-butoxybenzyl)-5-nitro-1*H*-benzo[d]imidazol-1-yl)-*N,N*-diethylethan-1-amine | UZZPOLCDCVWLAZ-UHFFFAOYSA-N | 2021 | 2024 |

Continued on next page

| Common name | Synonyms | IUPAC name | InChI keys | UNODC EWA | 1961 UN Convention |
| --- | --- | --- | --- | --- | --- |
| Isotodesnitazene | - | *N,N*-diethyl-2-(2-(4-isopropoxybenzyl)-1*H*-benzo[d]imidazol-1-yl)ethan-1-amine | KWIINCAJOQGWPO-UHFFFAOYSA-N | 2021 | - |
| *N*-desethyl etonitazene | - | 2-(2-(4-ethoxybenzyl)-5-nitro-1*H*-benzo[d]imidazol-1-yl)-*N*-ethylethan-1-amine | RESPFUMJVJRUMB-UHFFFAOYSA-N | 2021 | - |
| *N*-desethyl isotonitazene | Norisotonitazene | *N*-ethyl-2-(2-(4-isopropoxybenzyl)-5-nitro-1*H*-benzo[d]imidazol-1-yl)ethan-1-amine | YGIGEJHYUUWLMA-UHFFFAOYSA-N | 2021 | 2025 |
| *N*-piperidinyl etonitazene | Etonitazepipne, *N*-Piperidyl Etonitazene | 2-(4-ethoxybenzyl)-5-nitro-1-(2-(piperidin-1-yl)ethyl)-1*H*-benzo[d]imidazole | SHOWCAJXNDOKRF-UHFFFAOYSA-N | 2021 | 2025 |
| *N*-pyrrolidino etonitazene | Etonitazepyne | 2-(4-ethoxybenzyl)-5-nitro-1-(2-(pyrrolidin-1-yl)ethyl)-1*H*-benzo[d]imidazole | LQZWZCJCEPUKCJ-UHFFFAOYSA-N | 2021 | 2023 |
| 5-Methyl etodesnitazene | Etomethazene,  5-Methyl desnitroetonitazene,5-Methyl etazene | 2-(2-(4-ethoxybenzyl)-5-methyl-1*H*-benzo[d]imidazol-1-yl)-*N,N*-diethylethan-1-amine | UACYJENMCPODLZ-UHFFFAOYSA-N | 2022 | - |
| 6-Methyl etodesnitazene | 6-Methyl desnitroetonitazene | 2-(2-(4-ethoxybenzyl)-6-methyl-1*H*-benzo[d]imid azol-1-yl)-*N,N*-diethylethan-1-amine | XSIFQIYLBNMPGG-UHFFFAOYSA-N | 2022 | - |
| *N*-pyrrolidino protonitazene | Protonitazepyne | 5-nitro-2-(4-propoxybenzyl)-1-(2-(pyrrolidin-1-yl)ethyl)-1*H*-benzo[d]imidazole | KCRWXNIIXGBPID-UHFFFAOYSA-N | 2022 | 2025 |
| Ethyleneoxynitazene | Tetrahydrofuranitazene | 2-(2-((2,3-dihydrobenzofuran-5-yl)methyl)-5-nitro-1*H*-benzo[d]imidazol-1-yl)-*N,N*-diethylethan-1-amine | AREWSTCCFVRMMW-UHFFFAOYSA-N | 2023 | - |
| *Iso*-butonitazene | - | *N,N*-diethyl-2-(2-(4-isobutoxybenzyl)-5-nitro-1*H*-benzo[d]imidazol-1-yl)ethan-1-amine | DYOJYBAJCASQOS-UHFFFAOYSA-N | 2023 | - |
| *N*-desethyl protonitazene | - | *N*-ethyl-2-(5-nitro-2-(4-propoxybenzyl)-1*H*-benzo[d]imidazol-1-yl)ethan-1-amine | FRLXQVPRAVUYKW-UHFFFAOYSA-N | 2023 | - |
| *N,N-*dimethylamino etonitazene | *N,N*-dimethyl etonitazene | 2-(2-(4-ethoxybenzyl)-5-nitro-1*H*-benzo[d]imidazol-1-yl)-*N,N*-dimethylethan-1-amine | WUHIUQVAZXUZGB-UHFFFAOYSA-N | 2023 | - |
| *N*-pyrrolidino metonitazene | Metonitazepyne | 2-(4-methoxybenzyl)-5-nitro-1-(2-(pyrrolidin-1-yl)ethyl)-1*H*-benzo[d]imidazole | AIPDICOXJFKNKF-UHFFFAOYSA-N | 2023 | 2025 |

Continued on next page

| Common name | Synonyms | IUPAC name | InChI keys | UNODC EWA | 1961 UN Convention |
| --- | --- | --- | --- | --- | --- |
| Clodesnitazene | Desnitroclonitazene | 2-(2-(4-chlorobenzyl)-1*H*-benzo[d]imidazol-1-yl)-*N,N*-diethylethan-1-amine | AJUNJUWUDMYVRW-UHFFFAOYSA-N | 2024 | - |
| Fluetonitazene | F-Etonitazene | *N,N*-diethyl-2-(2-(4-(2-fluoroethoxy)benzyl)-5-nitro-1*H*-benzo[d]imidazol-1-yl)ethan-1-amine | LYIOWGDWEBHUGB-UHFFFAOYSA-N | 2024 | - |
| Methylenedioxynitazene | - | 2-(2-(benzo[d][1,3]dioxol-5-ylmethyl)-5-nitro-1*H*-benzo[d]imidazol-1-yl)-*N,N*-diethylethan-1-amine | UZYGPCHBXBCMHI-UHFFFAOYSA-N | 2024 | - |
| *N*-Pyrrolidino Fluetonitazene | Fluetonitazepyne, F-etonitazepyne | 2-(4-(2-fluoroethoxy)benzyl)-5-nitro-1-(2-(pyrrolidin-1-yl)ethyl)-1*H*-benzo[d]imidazole | ZTSPPQAICVWKPD-UHFFFAOYSA-N | 2024 | - |
| *N*-pyrrolidino isotonitazene | Isotonitazepyne | 2-(4-isopropoxybenzyl)-5-nitro-1-(2-(pyrrolidin-1-yl)ethyl)-1*H*-benzo[d]imidazole | XZGLLCLVIOGQGU-UHFFFAOYSA-N | 2024 | - |
| *N*-desethyl metonitazene | - | *N*-ethyl-2-(2-(4-methoxybenzyl)-5-nitro-1*H*-benzo[d]imidazol-1-yl)ethan-1-amine | LONAPKSOIKWYAV-UHFFFAOYSA-N | 2025 | - |
| Protodesnitazene | - | *N,N*-diethyl-2-(2-(4-propoxybenzyl)-1*H*-benzo[d]imidazol-1-yl)ethan-1-amine | WVMAKRNNNMYRNT-UHFFFAOYSA-N | 2025 | - |

**Table S1.** Nitazene compounds, showing their common name, synonyms, IUPAC names, the year of first reporting to the UNODC EWA, and, where applicable, the year of scheduling under the 1961 Single Convention on Narcotic Drugs [1–3].

**References**

1. United Nations Office on Drugs and Crime. Nitazenes reported to UNODC EWA over time. at <https://www.unodc.org/LSS/Announcement/Details/7e29daf9-1d49-45e6-95e7-8ce932bc94e1>

2. United Nations Office on Drugs and Crime. UNODC: CND decision on international control of four nitazenes enters into force. at <https://www.unodc.org/LSS/Announcement/Details/16bcac76-5c96-49f6-9d8a-56d1aa76cd7a>

3. United Nations Office on Drugs and Crime. International Drug Control Conventions. at <https://www.unodc.org/unodc/en/commissions/CND/Mandate_Functions/Scheduling.html>

**Figures**


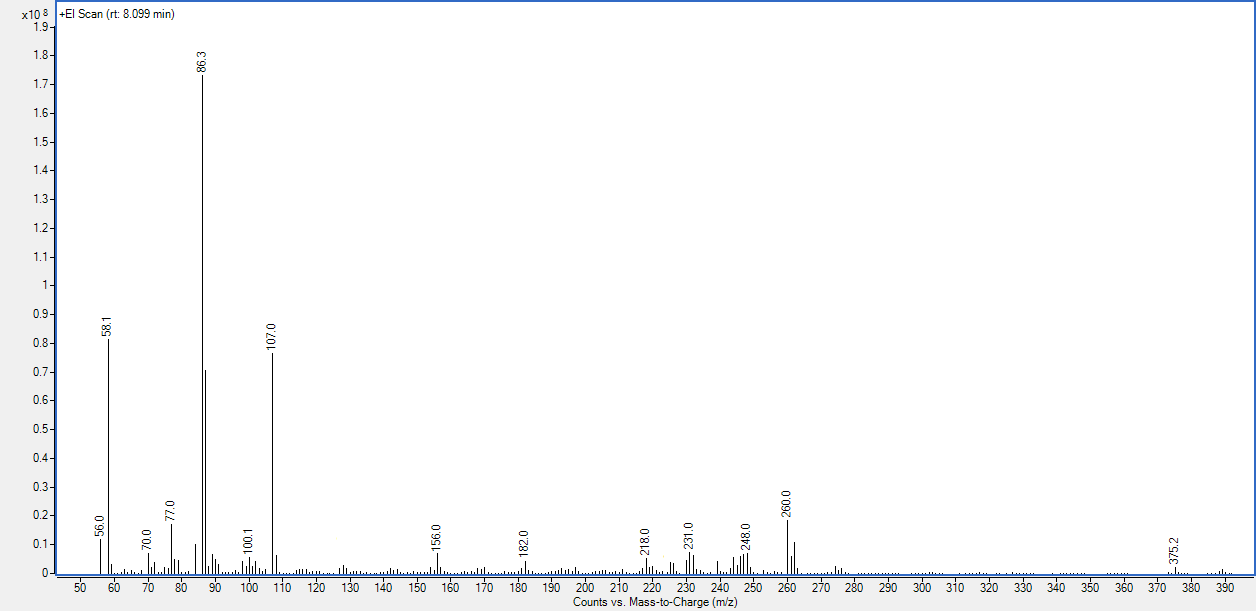


**Fig. S1.** GC-MS/MS mass spectrum of the nitazene sample peak at 8.099 min.


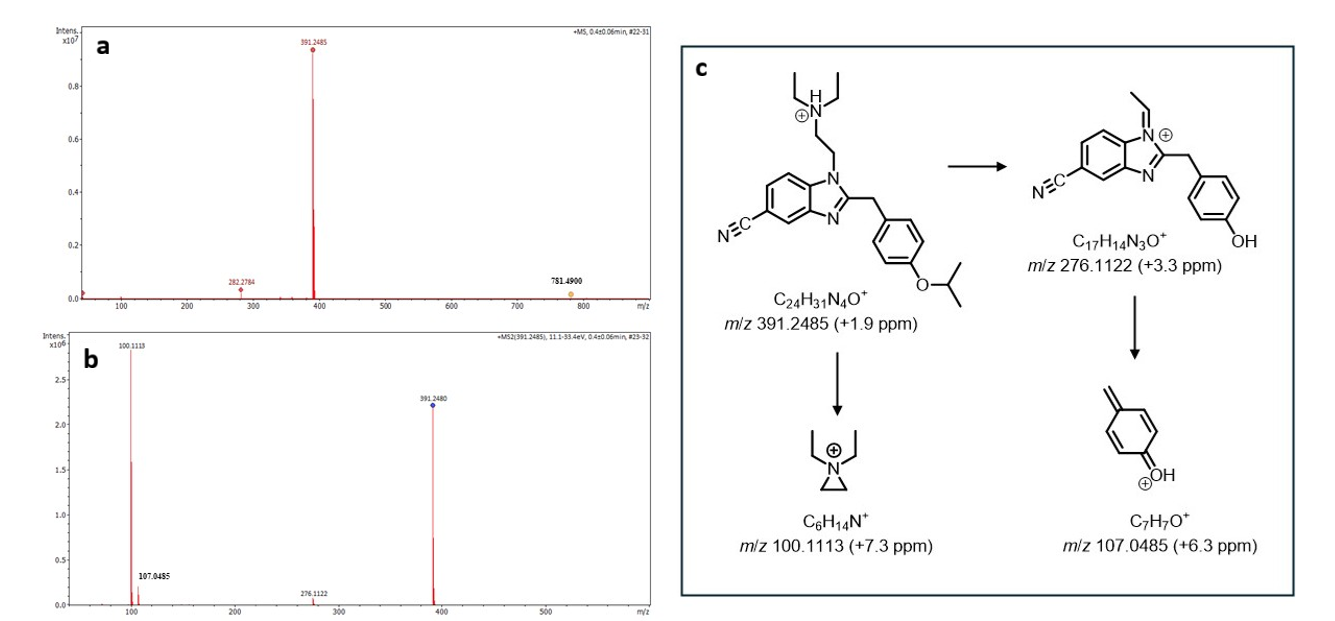


**Fig. S2.** HRMS data of 5-cyano isotodesnitazene: (**a**) full scan; (**b**) tandem mass spectrum; (**c**) proposed structures for the main fragment ions detected.


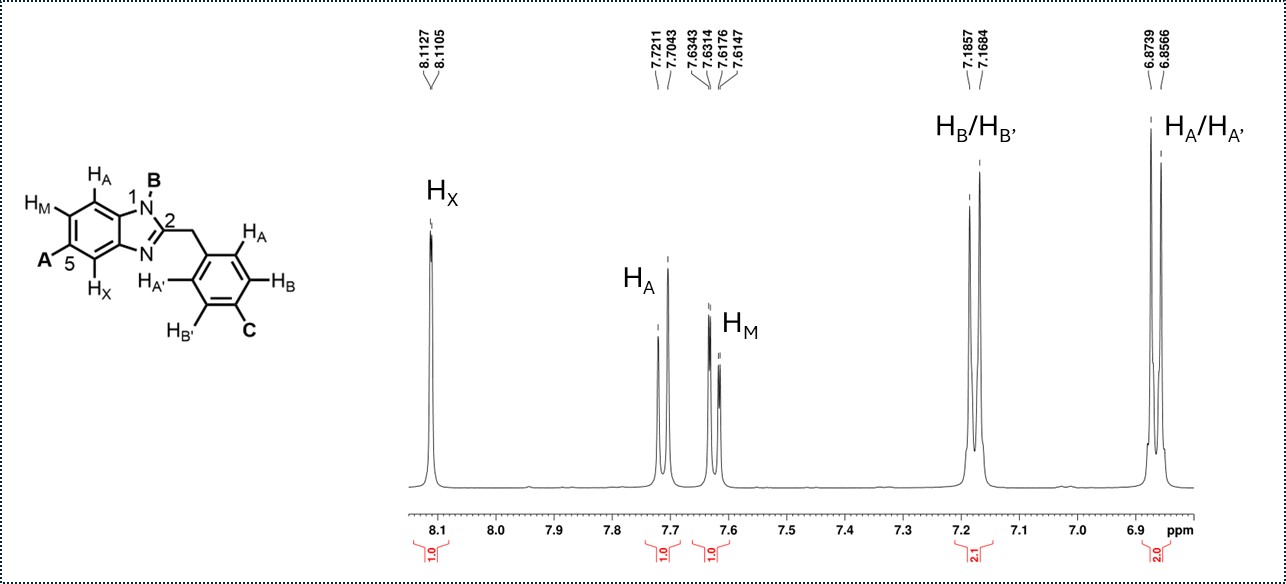


**Fig. S3.** ^1^H NMR (500 MHz, DMSO-*d_6_*) spectrum of the nitazene sample in the aromatic zone of the 2-benzylbenzimidazole moiety.


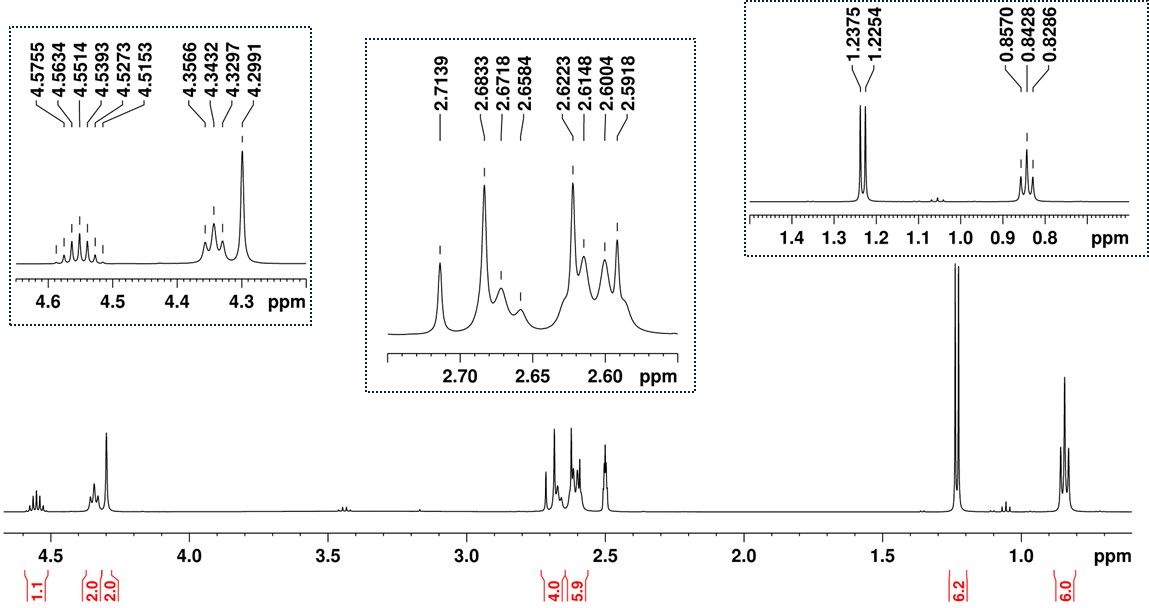


**Fig. S4.** ^1^H NMR (500 MHz, DMSO-*d_6_*) spectrum of the nitazene sample in the aliphatic region (4.67-0.60 ppm), with three in sets showing expanded areas of interest.


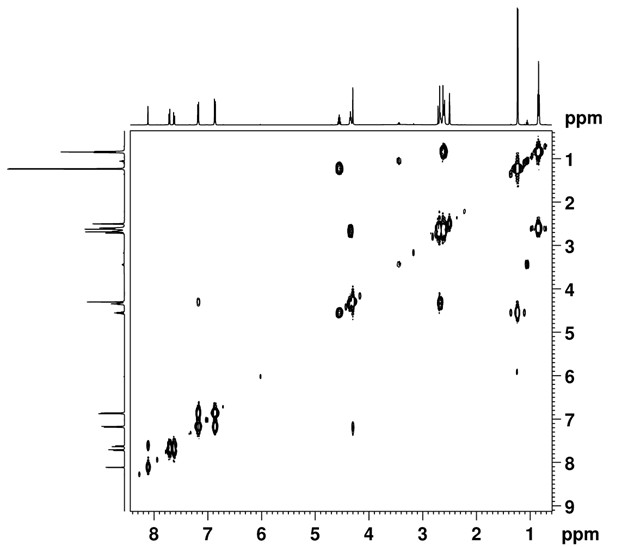


**Fig. S5.** [^1^H-^1^H]-COSY spectrum of the nitazene sample.


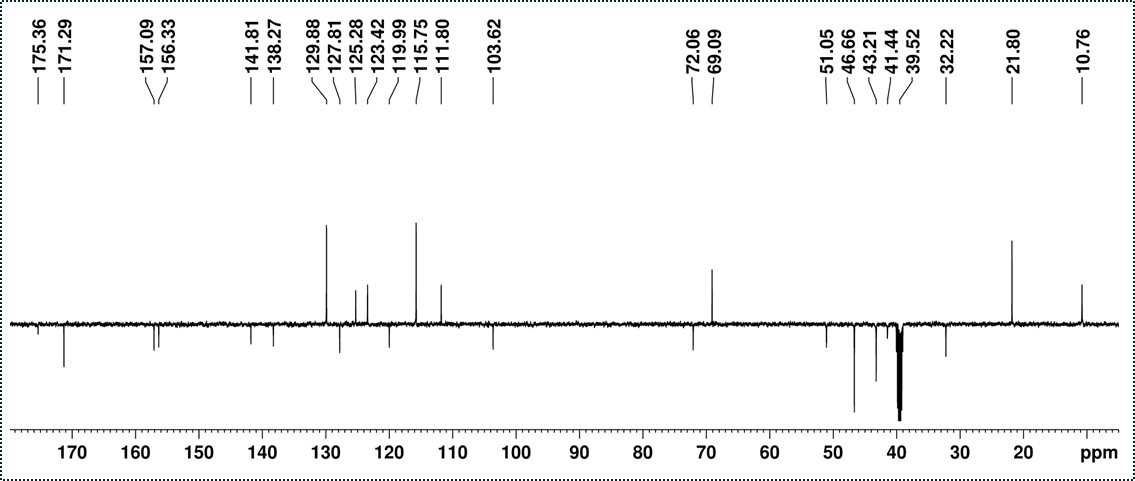


**Fig. S6.** ^13^C APT (125 MHz, DMSO-*d_6_*) spectrum of the nitazene sample.


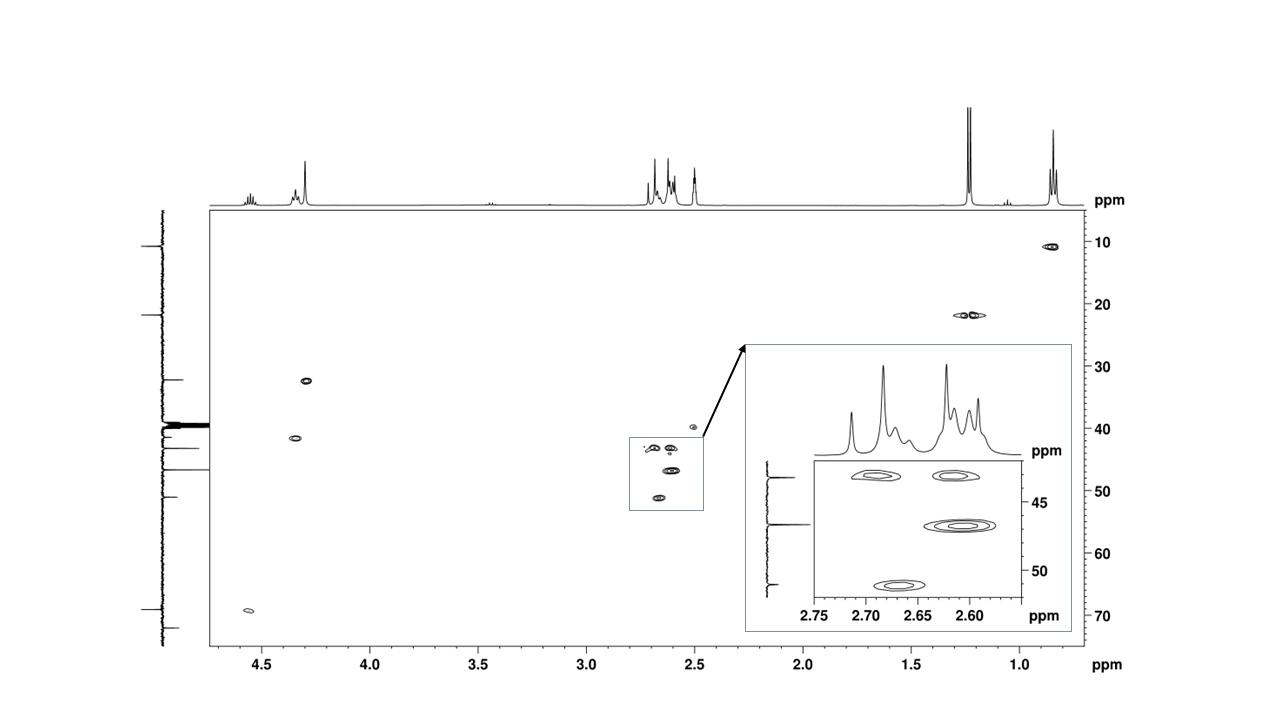


**Fig. S7.** [^1^H-^13^C]-HSQC spectrum (DMSO-*d_6_*) of the nitazene sample expanded in the aliphatic region (4.75-0.6 ppm) with one zooming zone (2.75-2.55 ppm).


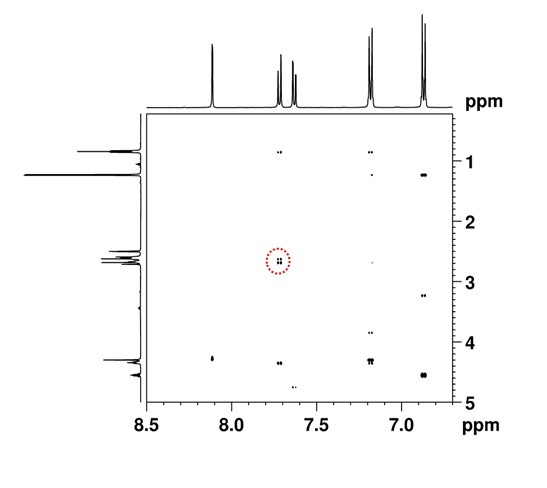


**Fig. S8.** [^1^H-^1^H]-NOESY spectrum of the nitazene sample, expanded in the region of the key correlations of aromatic protons (6.7-8.5 ppm) with aliphatic protons (0.5-5 ppm).
